# Causal association between basal metabolic rate and risk of cardiovascular diseases: a univariable and multivariable Mendelian randomization study

**DOI:** 10.1038/s41598-023-39551-2

**Published:** 2023-08-01

**Authors:** Yihua Li, Huiqi Zhai, Liang Kang, Qingmin Chu, Xinjun Zhao, Rong Li

**Affiliations:** 1 The First Clinical Medical College of Guangzhou University of Chinese Medicine, Guangzhou, Guangdong China; 2 Department of Cardiovascular Disease, The First Affiliated Hospital of Guangzhou University of Chinese Medicine, Guangzhou, Guangdong China

**Keywords:** Cardiology, Medical research, Risk factors

## Abstract

Basal metabolic rate (BMR) is associated with cardiovascular health; however, the causal relationship between BMR and the risk of cardiovascular diseases (CVDs) remains unclear. This study aimed to investigate the potential causal relationship of BMR on common CVDs including aortic aneurysm (AA), atrial fibrillation and flutter (AFF), calcific aortic valvular stenosis (CAVS), heart failure (HF), and myocardial infarction (MI) by Mendelian randomization (MR). The univariable MR analysis using inverse variance weighted (IVW) model as the primary analysis method revealed that genetically predicted higher BMR causally increased the risk of AA [IVW odds ratio (OR) = 1.34, 95% confidence interval CI  1.09–1.65, p = 0.00527], AFF (IVW OR = 1.87, 95% CI  1.65–2.12, p = 1.697 × E-22), and HF (IVW OR = 1.35, 95% CI  1.20–1.51, p = 2.364 × E-07), while causally decreasing the risk of MI (IVW OR = 0.83, 95% CI  0.73–0.93, p = 0.00255). In the multivariable MR analysis, which controlled for common cardiovascular risk factors, direct effects of BMR on an increased risk of AA and AFF, as well as a decreased risk of MI, but an attenuated causal effect on HF, were observed. In conclusion, the current MR study provides evidence for a causal relationship between BMR and the risk of AA, AFF, HF, and MI.

## Introduction

Cardiovascular diseases (CVDs) are global public health issues that threaten humanity, accounting for approximately one-third of all disease-related deaths^1^ and posing an increasing economic burden. The identification and treatment of risk factors are critical for the prevention of CVDs. Obesity, hypertension, hyperglycemia, and hyperlipidemia^2^ are reported as common cardiovascular risk factors that are also features of metabolic syndrome and manifestations of metabolic abnormalities.

Metabolism is a fundamental aspect of life. To maintain normal physiological functions, the human body uses nutrients obtained from food for energy metabolism. Changes in body weight can result from an imbalance between total energy intake and expenditure. Basal metabolic effect accounts for approximately 70% of daily energy expenditure^3^, the efficiency of which can be described as basal metabolic rate (BMR). Given its significance, BMR has received a lot of attention from health researchers and the general public. Lower BMR has been reported to be associated with an increased risk of obesity, insulin resistance, and type 2 diabetes (T2DM)^4–6^, implying that lower BMR is an undesirable factor for health. However, a higher BMR is also reported to be associated with a higher risk of multimorbidity in the elderly^7^, while increasing mortality^8^, suggesting that a higher BMR is a potential predictor of impending health deterioration.

Given the positive and negative impacts of BMR on health, its contribution to the risk of CVDs remains unclear. If we use heart failure (HF) as an example, observational studies have found an association between HF and higher BMR^9,10^, but opposing views have also been expressed^11^. Due to difficulty in completely excluding the effects of confounding factors and reverse causal associations in observational studies, the causal association between BMR and the risk of CVDs has not been well investigated in the literature.

Mendelian randomization (MR) is a useful and increasingly popular epidemiological approach that uses observational data to infer causality between risk factors and disease outcomes^12^. The basic idea underlying MR analysis is to use exposure-related genetic variants as instrumental variables (IVs) and evaluate the relationship between exposures and outcomes. MR analysis avoids the influence of confounding factors and reverse causality on causal inference, as genetic variants are randomly assigned during meiosis and genotypes are not altered by diseases^13^.

In this study, we use publicly available genome-wide association study (GWAS) data to conduct univariable MR (UVMR) analysis to investigate the potential causality between BMR and CVDs. Subsequently, we performed multivariable MR (MVMR) analysis adjusting for common cardiovascular risk factors including body mass index (BMI), T2DM, blood pressure, and smoking to assess the direct effect of BMR on CVDs.

## Materials and methods

### Data sources

The GWAS summary data of BMR in Europeans were obtained from the IEU Open GWAS database (https://gwas.mrcieu.ac.uk/), which was an analysis of the results of the UK Biobank^14^ phenotypes survey conducted by the Medical Research Council-Integrative Epidemiology Unit (MRC-IEU) consortium^15,16^, including 9,851,867 single nucleotide polymorphisms (SNPs) from 454,874 participants. BMR was measured using bioelectric impedance analysis conducted by the BC-418 body composition analyzer (manufactured by TANITA in Tokyo, Japan), depending on the weight, age, gender, and fat-free mass of the participants^17,18^.

BMR was used as an exposure variable in this MR study, and five cardiovascular phenotypes were used as outcomes variables: aortic aneurysm (AA), atrial fibrillation and flutter (AFF), calcific aortic valvular stenosis (CAVS), HF, and myocardial infarction (MI). The GWAS data of CVDs were derived from the FINNGEN^19^, a large public biobank aiming to collect and analyze genome and health data from 500,000 Finnish participants. The latest GWAS data including 16,962,023 variants from 309,154 participants were released publicly via FinnGen Release 7 on June 1st, 2022.

In addition, GWAS data for common cardiovascular risk factors, including BMI^20^, T2DM^21^, smoking initiation (SI)^22^, systolic blood pressure (SBP), and diastolic blood pressure (DBP), used in the MVMR analysis were obtained through the MRC-IEU^15,16^. Detailed source information for the GWAS data is provided in Table 1.

**Table 1:** Detailed information for the GWAS data.

Phenotypes	Consortium	ID	Participants	Population
BMR	MRC-IEU	ukb-b-16446	454,874	European
BMI	MRC-IEU	ebi-a-GCST006368	315,347	European
T2DM	MRC-IEU	ebi-a-GCST006867	655,666	European
SI	GSCAN	ieu-b-4877	607,291	European
SBP	MRC-IEU	ukb-b-20175	436,419	European
DBP	MRC-IEU	ukb-b-7992	436,424	European
AA	FinnGen	I9_AORTANEUR	294,730	European
AFF	FinnGen	I9_AF	191,205	European
CAVS	FinnGen	I9_CAVS	309,154	European
HF	FinnGen	I9_HEARTFAIL	292,047	European
MI	FinnGen	I9_MI	280,528	European

*BMR* basal metabolic rate, *BMI* body mass index, *T2DM* type 2 diabetes, *SI* smoking initiation, *SBP* systolic blood pressure, *DBP* diastolic blood pressure, *AA* aortic aneurysm, *AFF* atrial fibrillation and flutter, *CAVS* calcific aortic valvular stenosis, *HF* heart failure, *MI* myocardial infarction.

### Selection for IVs

The SNPs selected as IVs for BMR were obtained by running the function “*extract_instruments*” of the R package “*TwoSampleMR*”. According to previous studies, the IVs selected for exposure used in MR analysis should meet the criteria of *p* < 5 × E-08 and linkage disequilibrium (LD) *r*^2^ < 0.001 within 10 mb^23^. SNPs were extracted from the summary statistics of the five cardiovascular outcomes, whereas those associated with outcomes with genome-wide significance (*p* < 5 × E-08) were discarded. The palindromic SNPs with intermediate allele frequencies were then removed using the "*harmonise data*" function. Subsequently, SNPs were uploaded to the PhenoScanner database^24,25^ to find and remove those significantly associated with common cardiovascular risk factors (including BMI or obesity, diabetes, smoking, and blood pressure) to reduce the effect of confounding factors (Supplementary Tables S1). Then, the “*run_mr_presso*” function (set *NbDistribution* = 10,000, *SignifThreshold* = 0.05) was used for detecting horizontal pleiotropy^26^, and the outlier variants were removed when a significant horizontal pleiotropy was detected. Finally, the *F* statistics of the remaining SNPs used in UVMR were calculated using the following formula: *F* = R^2^ × (N–k–1)/[(1–R^2^) × k], where R^2^ = 2 × *β*^2^ × (1–EAF) × EAF, N is the sample size of BMR, k is the number of SNPs, *β* is the estimate of genetic effect on BMR, and EAF is the frequency of the effect allele. The SNPs with an *F* statistic greater than 10 were considered strong IVs of BMR^27^.

### Statistical analysis

The inverse variance weighted (IVW) method was used as the primary analysis for the UVMR analysis, with MR-Egger, MR pleiotropy residual sum and outlier (MR-PRESSO), and weighted median (WM) model as supplemental ones. The IVW method can effectively combine the Wald ratio from multiple valid IVs into a single causal estimate for a more precise estimate of causal effect^28^. If heterogeneity is present, we employed the inverse-variance weighted (IVW) method with a random effects model for analysis, aiming to obtain more conservative outcomes^29^. When pleiotropy of IVs exists, MR-Egger is a valuable tool for estimating the causal effect^30^. The MR-PRESSO test can identify and remove any outlier variants, thus obtaining outlier-corrected MR analysis results, which are applicable when < 50% of the IVs are detected with horizontal pleiotropy^26^. The WM allows up to 50% of the IVs to be invalid and still produce consistent results^31^. MR-Egger and WM can provide more robust results but with less efficiency, while their consistency with the direction of IVW can increase confidence in estimating causal effects^32^. Given that five exposures and four analysis methods were included in this study, we consider a significant causal effect at the threshold of *p* < 0.0025 (Bonferroni correction *p* = 0.05/20) and nominally significant results at *p* < 0.05.

Following UVMR estimates, we conducted a sensitivity analysis to detect potential heterogeneity and pleiotropy and identify any violations of the basic assumptions of MR. The presence of heterogeneity and pleiotropy can weaken and even invalidate the causal inference process and results. Therefore, we additionally conducted Cochran’s Q-test and MR-Egger intercept analysis. For Cochran’s Q-test, a *p*-value of < 0.05 indicates the presence of heterogeneity. Further, for MR-Egger intercept analysis, a *p*-value of < 0.05 signifies the presence of pleiotropy^33^. Besides, the leave-one-out (LOO) analysis can be used to test the reliability and robustness of the MR analysis. The consistency of LOO analysis and IVW results suggests that the MR analysis process and causal inference results are reliable and robust.

Considering the potentially confounding or mediating effects of common cardiovascular risks on the results of MR analysis, we performed MVMR analysis, adjusting for BMI, T2DM, SI, SBP, and DBP, to obtain direct effects of BMR on CVDs as well as to assess possible mediating effects. Similarly, we used the criteria of *p* < 5 × E-08 and linkage disequilibrium (LD) r^2^ < 0.01 within 10 mb^23^ to filter IVs and subsequently removed those significantly associated with outcomes. For the MVMR analysis, we used IVW, WM, and MR-Egger methods as the main analysis approaches. The WM model was superior to the IVW model in detecting heterogeneity because it naturally explained heterogeneity by the bootstrapped variance. In addition, pleiotropy was evaluated by the Egger regression intercept, and a *p* < 0.05 indicated the presence of pleiotropy.

MR estimates and sensitivity analyses were performed using R packages “*TwoSampleMR* (version 0.5.6)”^16^ and “*MendelianRandomization* (version 0.3.0)”^34^ in R (version 4.2.1). On the other hand, we leveraged an online program (https://shiny.cnsgenomics.com/mRnd/) launched by Brion et al.^35^ to calculate statistical power (recommended to be > 80%).

## Results

### UVMR estimates

For each outcome, a different number of SNPs were filtered and chosen as IVs for causal inference (Supplementary Tables S2–S6). The cumulative R^2^ and F-statistics of IVs ranged from 0.0217 to 0.0230 and 9885.34 to 10,458.51, respectively, indicating that the IVs used were sufficiently robust (Supplementary Tables S2–S6). Additionally, statistical power estimates are shown in Supplementary Table S7.

**Figure 1:**
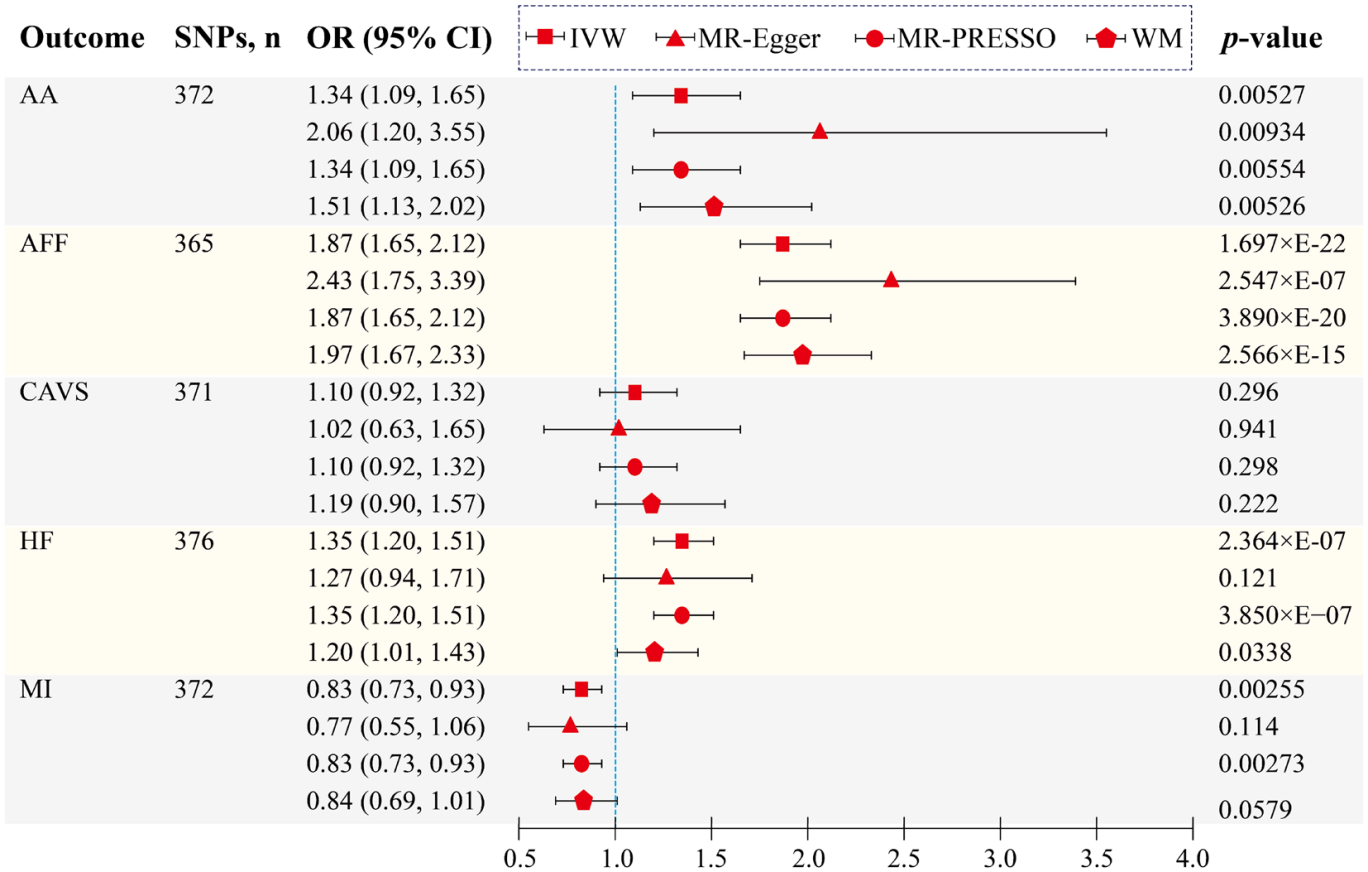
UVMR analysis of the causal relationship between BMR and CVDs. *SNPs* single-nucleotide polymorphisms, *OR* odds ratio, *CVDs* cardiovascular diseases, *CI* confidence interval, *IVW* inverse variance weighted, *MR*-*PRESSO* MR pleiotropy residual sum and outlier, *WM* weighted median, *BMR* basal metabolic rate, *AA* aortic aneurysm, *AFF* atrial fibrillation and flutter, *CAVS* calcific aortic valvular stenosis, *HF* heart failure, *MI* myocardial infarction.

**Figure 2:**
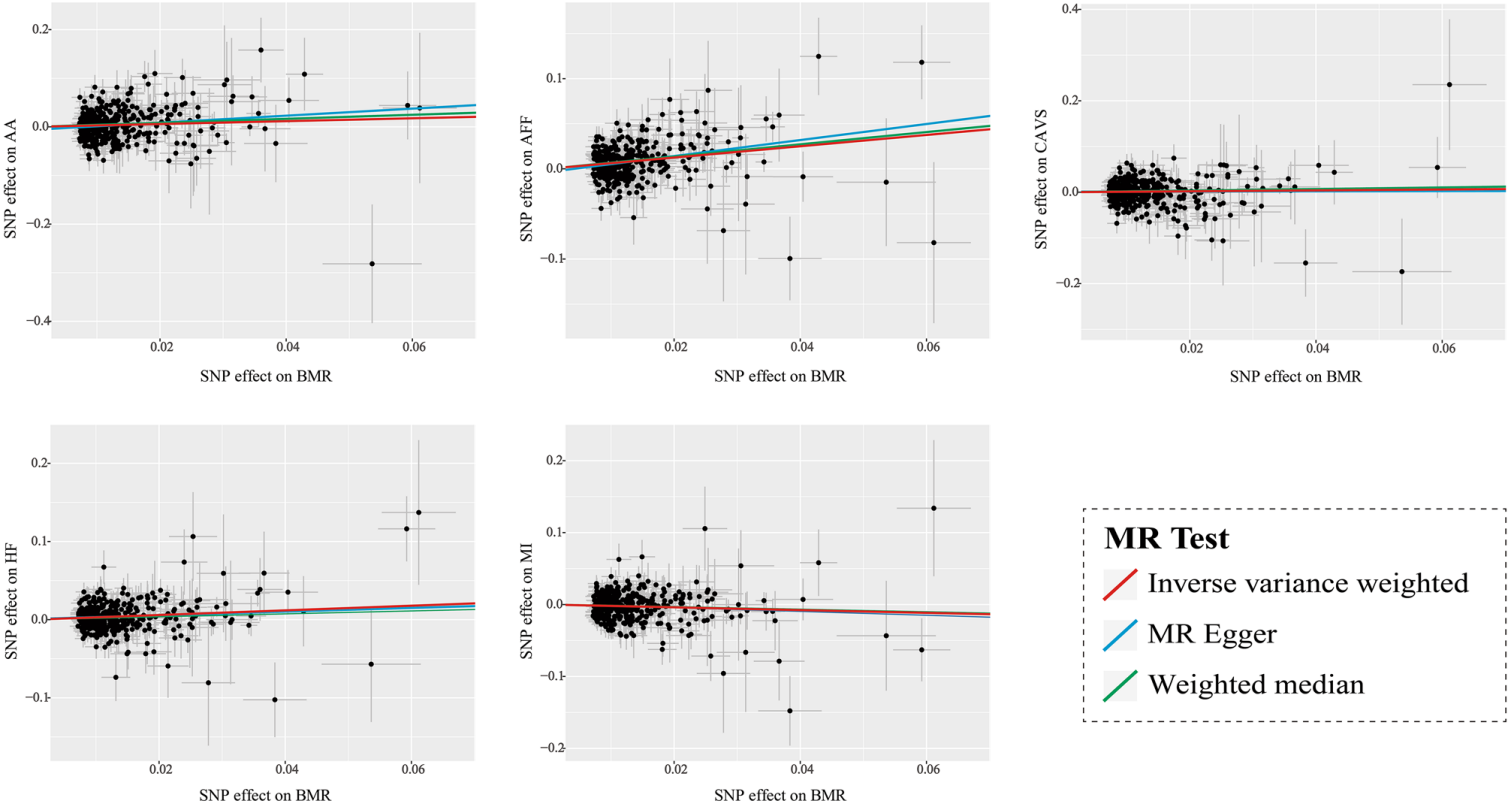
Scatter plots visualize the causal effects of BMR on CVDs. *BMR* basal metabolic rate, *CVDs* cardiovascular diseases, *AA* aortic aneurysm, *AFF* atrial fibrillation and flutter, *CAVS* calcific aortic valvular stenosis, *HF* heart failure, *MI* myocardial infarction.

After removing outliers detected by MR-PRESSO analysis (Supplementary Tables S8), genetically predicted higher BMR showed significant causal effects on the risks of three cardiovascular outcomes, as revealed by the IVW model. The findings of the present study provide evidence indicating that higher BMR increases the risk of AA [IVW odds ratio (OR) = 1.34, 95% confidence interval CI   1.09–1.65, p = 0.00527, nominally significant], AFF (IVW OR = 1.87, 95% CI  1.65–2.12, p = 1.697 × E-22), and HF (IVW OR = 1.35, 95% CI  1.20–1.51, p = 2.364 × E-07), as well as causally decreases the risk of MI (IVW OR = 0.83, 95% CI  0.73–0.93, p = 0.00255, nominally significant). Further, this study does not support the causality of BMR on the risk of CAVS (Fig. 1, Supplementary Table S9). Causal inferences were broadly similar among different MR methods, which was visualized in the scatter plots (Fig. 2). After removing the outliers, the MR-PRESSO distortion test revealed no significant change in the results of causal inference between BMR and the five cardiovascular phenotypes (*p* > 0.05) (Supplementary Table S8).

Regarding sensitivity analysis, Cochran’s-Q-test results indicated heterogeneity in the present UVMR analysis (*p* < 0.05), but Egger regression did not detect any horizontal pleiotropy, as all Egger-intercepts had *p* > 0.05 (Supplementary Table S9). LOO analysis (Supplementary Figs. S1–S5) revealed that no individual SNPs drives the results of causal inference between BMR and the five cardiovascular disorders. Additionally, the funnel plots were symmetrical, indicating that MR analysis results were robust and reliable (Supplementary Fig. S6).

### MVMR estimates

Since heterogeneity was detected during the MVMR analysis, the results of the WM model were given priority in the interpretation of causal inferences. In addition, Egger intercept analysis indicated the absence of any horizontal pleiotropy (Supplementary Table S10).

**Figure 3:**
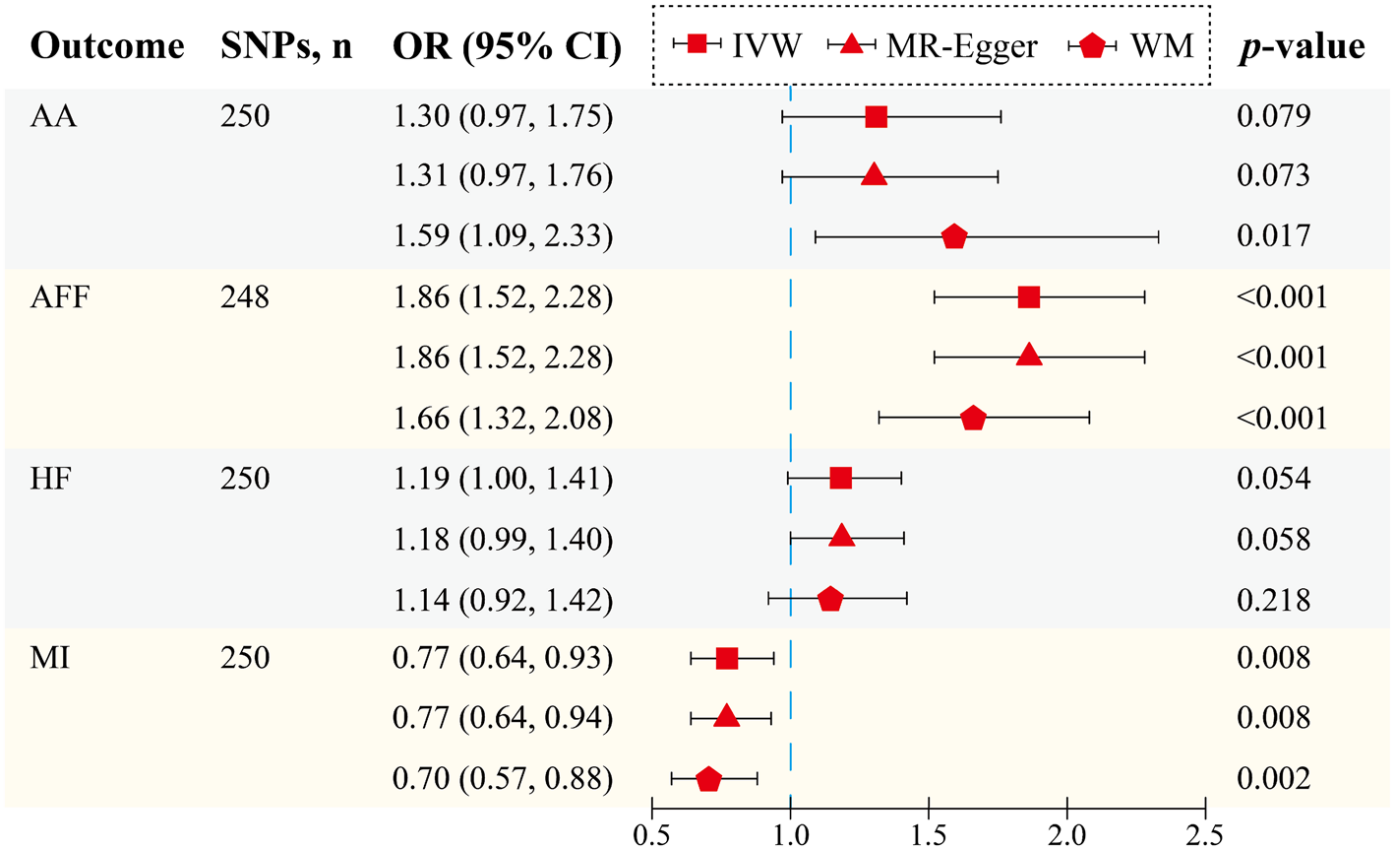
MVMR analysis of the effect of BMR on AA, AFF, HF, and MI, after adjusting for BMI, T2DM, SI, SBP, and DBP. *SNPs* single-nucleotide polymorphisms, *OR* odds ratio, *CI* confidence interval, *IVW* inverse variance weighted, *WM* weighted median, *AA* aortic aneurysm, *AFF* atrial fibrillation and flutter, *HF* heart failure, *MI* myocardial infarction.

There is strong evidence to support a direct causal effect of BMR on an increased risk of AFF (WM OR = 1.66, 95% CI  1.32–2.08, *p* < 0.001) and a decreased risk of MI (WM OR = 0.70, 95% CI  0.57–0.88, *p* = 0.002) in MVMR analysis that controls for cardiovascular risk factors, including BMI, T2DM, SI, SBP, and DBP. This was also consistent with the assessment of the causal effect of BMR on the risk of AFF and MI derived independently from the IVW, and MR-Egger models. Furthermore, the results obtained by the WM method showed that the estimated causal effect of genetically predicted BMR on an increased risk of AA (WM OR = 1.59, 95% CI  1.09–2.33, *p* = 0.017) remained significant even after adjusting for cardiovascular risk factors. However, after controlling for cardiovascular risk factors, the causal effect of genetically predicted BMR on an increased risk of HF (WM OR = 1.14, 95% CI  0.92–1.42, *p* = 0.218) was attenuated (Fig. 3, Supplementary Table S10).

## Discussion

To the best of our knowledge, this is the first study to apply UVMR and MVMR to determine the causal association between BMR and the risk of CVDs. We found that genetically predicted higher BMR causally increased the risk of AA, AFF, and HF, as well as decreased the risk of MI. Moreover, the pattern of causal associations of BMR and the risk of AA, AFF, and MI remained even after adjusting for common cardiovascular risk factors but was attenuated for the risk of HF.

Until now, there have been limited studies examining the link between BMR and the risk of CVDs. Delaney C et al. reported that patients with abdominal AA had higher daily resting energy expenditure than their age- and sex-matched controls, which they speculated to be associated with the long-term exposure of patients to inflammatory diseases^36^. Patients with atrial fibrillation (AF) also had higher BMR, and those with permanent AF had a higher BMR than those with persistent or paroxysmal AF^37^. Further, some studies reported that higher BMR was observed among patients with HF^9,10^, whereas Zampino et al. reported that incident HF was associated with a steeper decline in BMR^11^. However, although the above-mentioned studies showed an association between BMR and AA, AF, and HF, a causal effect could not be determined. This is now complemented by our present research findings, wherein MR analysis revealed the role of higher BMR as a causal risk factor in the development of AA, AFF, and HF. Interestingly, we also observed a causal relationship between higher BMR and a reduced risk of MI, which was not previously reported.

The mechanism by which elevated BMR leads to an increased risk of AA, AFF, and HF remains unclear, and the following speculations may help elucidate the underlying mechanism. The BMR data used in the present study was acquired using the Body Composition Analyzer (BC-418), the operating principle of which is based on an improved recursion formula constructed by the combination of resting respiratory metabolism and fat-free mass (FFM)^17,18^. FFM is a critical factor influencing BMR, and people with higher BMR tend to have higher FFM. Previous MR studies have reported a causal association between higher FFM and an increased risk of AF^38^ and abdominal AA^39^. Numerous studies have found that FFM is a major risk factor for AF^40–42^. In addition, FFM was reported as a robust independent correlate of increased LV mass^43^, which may partially explain the increased risk of HF^44^. From another perspective, an elevated BMR implies that the body needs to produce more energy, which depends on the mitochondrial oxidative phosphorylation process, but this would also increase the production of reactive oxygen species (ROS), according to the perspective of the mitochondrial theory of aging^45,46^. Abnormal accumulation of ROS triggers oxidative stress and damages mitochondrial DNA, causing mitochondrial dysfunction and further increasing ROS accumulation, thereby forming a vicious cycle^47^ and increasing the risk of cardiovascular disease. Moreover, mitochondrial dysfunction results in increased BMR, which may be due to the need for increased nutrient oxidation to maintain cellular energy levels^48^. In addition, the renin-angiotensin system (RAS) and sympathetic nervous system (SNS) play significant and extensive roles in the development of CVD. The brain RAS has been reported to have a controlling effect on energy expenditure. Activation of RAS can increase energy consumption by increasing BMR^49,50^^.^ Besides, SNS plays a vital role in regulating energy consumption. SNS overactivity increases ATP consumption, heart rate, and blood pressure, leading to elevated BMR^51,52^. We consider that RAS and SNS may be essential bridges in deciphering the relationship between BMR and the risk of CVD.

On the other hand, the surprising finding of this study is that genetic predicted higher BMR causally decreases the risk of MI, which has not been previously described. However, it is difficult to explain this causal association because of the lack of relevant researches. Given that the primary pathological basis of MI is atherosclerosis, which differs from AA, AFF, and HF, we posit that an exploration of the relationship between BMR and atherosclerosis can provide insights into interpreting the finding of this study. A prospective study by B Sezgin et al.^53^ found that one of the most significant reasons for subclinical atherosclerosis in postmenopausal women may be the decrease in BMR. Additionally, research by Michael J. Toth et al.^54^ showed that males with higher resting energy expenditure have lower levels of body fat and better lipid metabolism, which may partially explain the protective effect of high BMR on MI.

Multiple observational studies have shown that BMR gradually decreases with age^54,55^. However, can we conclude that the age-related decrease in BMR will reduce the risk of AFF, AA, and HF while increasing the risk of MI? Obviously, the answer is in the negative. The age-related decrease in BMR may be due to changes in body composition, such as a decrease in fat-free mass (FFM)^55^, and a decrease in metabolic demands, which is an adaptive change in the body. If BMR remains inappropriately high during the aging process, it may be a predictor of deteriorating health^8,56^. This inappropriate increase in BMR may be the body's need to increase additional energy expenditure to repair or maintain homeostasis in response to imbalances caused by disease states^57^. Additionally, as mentioned earlier, the inappropriate decrease in BMR is one of the possible reasons for increased MI risk. Therefore, we should recognize that the age-related decrease in BMR is a normal physiological change, and it is the sustained abnormal levels of BMR, including both increase and decrease, that should be of concern.

The findings of this MR study may provide valuable insights for further research and public health considerations regarding cardiovascular diseases and BMR in the context of human aging. Firstly, based on the results of this study, BMR is not a simple risk factor or protective factor for cardiovascular health, but rather a comprehensive indicator of cardiovascular health. Secondly, abnormal BMR results may indicate the presence of cardiovascular health risks and suggest the need to lower cardiovascular health risks by identifying and correcting overall metabolic abnormalities in the body.

The present study has several significant strengths. First, we have explored the relationship between BMR and CVDs using MR analysis and have determined that higher BMR can causally increase the risk of AA, AFF, and HF, as well as decrease the risk of MI. The advent of bioelectric impedance analysis (BIA) now enables us to measure BMR quickly and accurately^58^, making it possible to use BMR as an easily applicable detector of the risk of AA, AFF, HF, and MI. Second, the use of MR essentially reduced the influence of confounding factors on the study results, and the absence of pleiotropy in the MR analysis enhanced our confidence in causal inference. In addition, considering common cardiovascular risk factors, the use of MVMR allowed us to find an independent effect of BMR on the risk of AA, AFF, and MI^59,60^. Finally, all the GWAS data in this study were restricted to European ancestry, which reduces bias from ethnic stratification, although this may limit the generalization of the findings to other populations. Previous studies have shown differences in BMR between different populations^61–63^. These differences in BMR may contribute to variations in the likelihood of obesity and could be one of the reasons for differences in the incidence of cardiovascular diseases among different populations^64^. However, there is currently no direct comparative research on the association between BMR and cardiovascular diseases in different populations. Therefore, in the future, it would be valuable to investigate the relationship between BMR and the risk of CVDs across different populations.

This study also has some other limitations. Firstly, the mechanistic explanations for the BMR-related increase in the risk of AA, AFF, and HF, as well as the BMR-related decrease in MI in our study, are speculative, making it challenging to explain the discordant associations. Secondly, BMR differed by gender and age, but performing further stratified analysis was challenging due to the use of summary-level GWAS data in this study. Moreover, as mentioned earlier, the BMR data used in this study were measured using BIA method, which has the advantages of being convenient, non-invasive, and having good repeatability^65^. However, since the principle of BIA for measuring BMR relies on the different electrical properties of tissues, it can be influenced by factors such as fluid retention, diet, diuretic use, and physical activity^66^. This may introduce measurement biases to our study. Future research could focus on studying the mechanisms by which BMR affects cardiovascular health and conducting broader clinical observational studies to facilitate the clinical translation of BMR as a cardiovascular health predictor.

## Conclusions

Our MR study provides evidence that higher BMR causally increases the risk of AA, AFF, and HF, while reducing the risk of MI. It also reveals that BMR has a direct impact on the increased risk of AA and AFF and the decreased risk of MI, independent of common cardiovascular risk factors.

## Supplementary Information

Supplementary Figure S1.

Supplementary Figure S2.

Supplementary Figure S3.

Supplementary Figure S4.

Supplementary Figure S5.

Supplementary Figure S6.

Supplementary Tables.

## Data Availability

All GWAS data used in our study are publicly available.
