# Supplementary figures and images for: Causal association between basal metabolic rate and risk of cardiovascular diseases: a univariable and multivariable Mendelian randomization study
Source: Sci Rep. 2023 Aug 1;13:12487. doi: 10.1038/s41598-023-39551-2 (PMC10393961; doi:10.1038/s41598-023-39551-2)

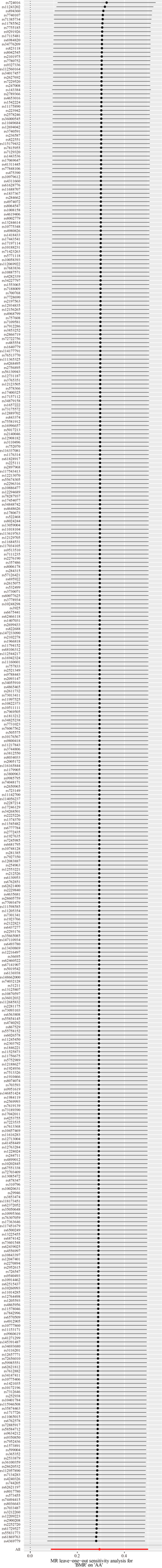

Supplement: Supplementary file 1 — Supplementary Figure S1. [file 41598_2023_39551_MOESM1_ESM.jpg]

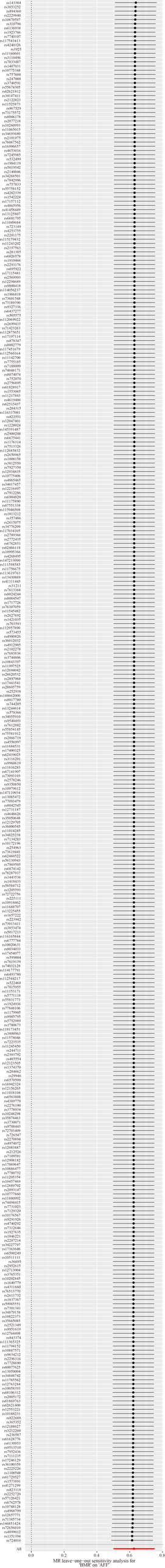

Supplement: Supplementary file 2 — Supplementary Figure S2. [file 41598_2023_39551_MOESM2_ESM.jpg]

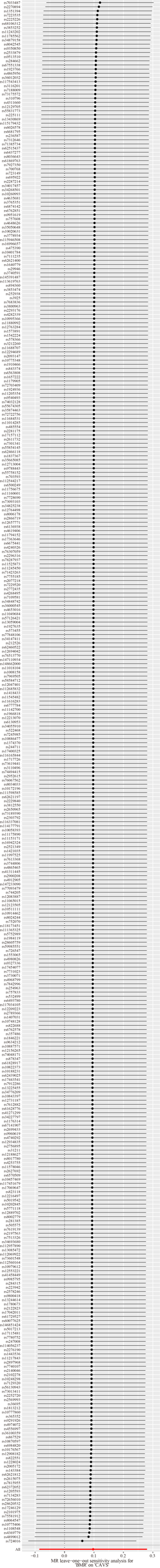

Supplement: Supplementary file 3 — Supplementary Figure S3. [file 41598_2023_39551_MOESM3_ESM.jpg]

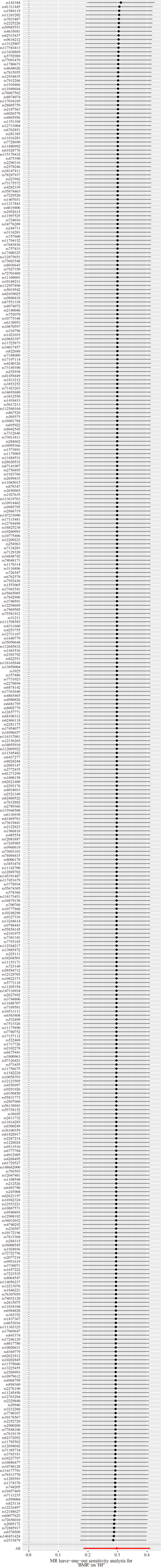

Supplement: Supplementary file 4 — Supplementary Figure S4. [file 41598_2023_39551_MOESM4_ESM.jpg]

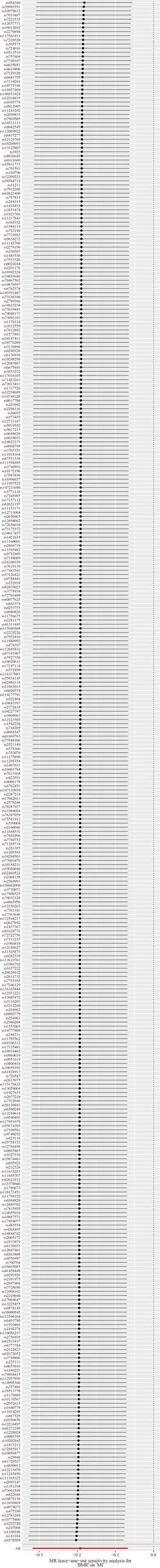

Supplement: Supplementary file 5 — Supplementary Figure S5. [file 41598_2023_39551_MOESM5_ESM.jpg]

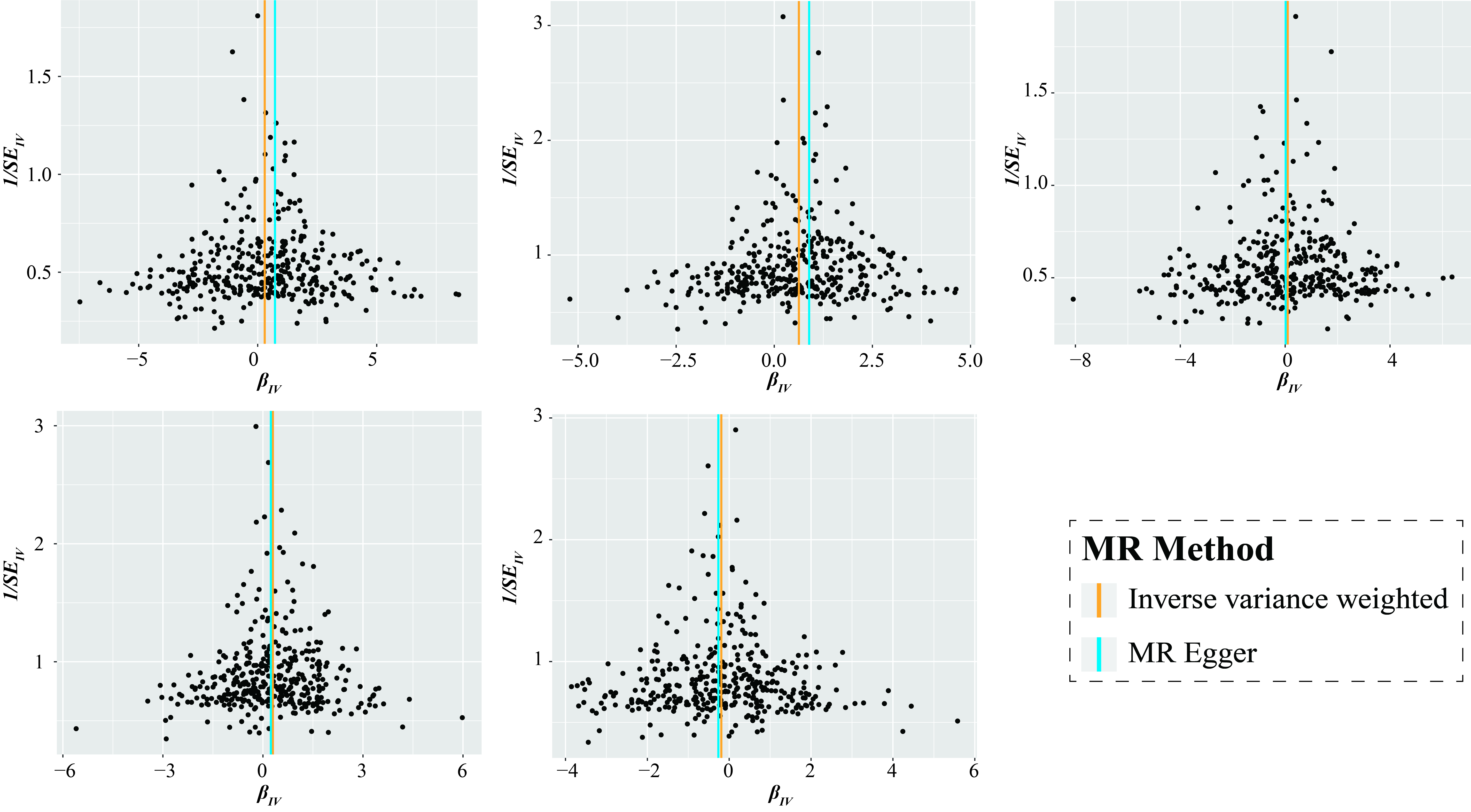

Supplement: Supplementary file 6 — Supplementary Figure S6. [file 41598_2023_39551_MOESM6_ESM.jpg]
